# The Prevalence of Behçet’s Disease in a Thrombosis Clinic

**DOI:** 10.31138/mjr.34.1.66

**Published:** 2023-02-21

**Authors:** Mira Merashli, Paul RJ Ames

**Affiliations:** 1 Department of Internal Medicine, Rheumatology Division, American University of Beirut, Beirut, Lebanon,; 2 CEDOC, NOVA Medical School, Faculdade de Ciências Médicas, Universidade NOVA de Lisboa, Lisboa, Portugal,; 3 Dumfries & Galloway Royal Infirmary, Dumfries, Scotland, United Kingdom

**Keywords:** Behçet’s disease, prevalence, thrombosis clinic

## Abstract

**Background:**

the prevalence of venous thromboembolism (VTE) in Behcet’s disease (BD) is around 40%, though recognition of BD in a thrombosis clinic has been poorly addressed.

**Objective:**

to evaluate the prevalence of signs and symptoms leading to the diagnosis of BD in a thrombosis clinic compared to patients attending a general haematology clinic and to healthy controls. Design: cross-sectional case-double control anonymous questionnaire survey. Participants: consecutive patients with spontaneous VTE (n=97) attending a thrombosis clinic, consecutive patients from a general haematology (GH) clinic (n=89) and controls (CTR).

**Results:**

BD was diagnosed in 1.03% of VTE participants, in 2.2% of GH participants and in 1.2% of healthy CTR. Exhaustion was more common reported in participants from the VTE group (15.6%) than in those from the GH group (10.3%) and from the healthy CTR (3%) (p=0.06); the sum of signs and symptoms of BD clustered in the VTE group (89.5%) compared to the GH (72.4%) and the CTR (59.7%) (p<0.0001).

**Conclusions:**

BD may be diagnosed in 1 every 100 patients with VTE attending a thrombosis clinic and in 2 every 100 patients attending a GH clinic: awareness must be raised not to under-diagnose or misdiagnose BD in these settings as management of VTE in BD deviates from the norm.

## INTRODUCTION

Behçet’s disease (BD) is a systemic vasculitis characterized by recurrent oral and genital aphthosis, ophthalmic, cutaneous, articular, intestinal, urogenital, neurological, pulmonary and vascular manifestations.^1^ The prevalence of BD in the Western countries is approximately 1 per 100,000 according to geographical area with rates of 0.5–3 per 100,000 reported in Europe^2^ the UK prevalence is around 5 per 100,000 inhabitants as calculated from the Hertfordshire area.^3^ The prevalence of vascular involvement may be as high as 40% according to the ethnicity of the population under study^4^ and superficial thrombophlebitis accompanies vascular occlusion in almost 13% of patients.^5^ The vascular manifestations are mostly venous thromboembolism (VTE) occurring mostly in lower and upper lower limbs, in the superior and inferior vena cava, in the supra-hepatic vein and in the cerebral veins with prevalence ranging between 3% and 41%.^5^

Two UK surveys reported a thrombosis prevalence of 32% in BD^6,7^; given this high prevalence of vessel involvement we performed a questionnaire survey of symptoms and signs of BD to assess whether it was under-recognised in patients attending a thrombosis clinic and the results are presented herein.

## MATERIALS AND METHODS

### Objective

To estimate the prevalence of BD disease in a thrombosis clinic and compare it to the prevalence derived from a general haematology clinic and from healthy controls.

### Participants

Survey design: retrospective cross-sectional case-double control. During the period February 2021 - July 2021 a total of 349 consecutive patients (265 with venous occlusions and 84 with pulmonary embolism) were referred from the accident and emergency department to the anticoagulant clinic; of these 194 (155 with venous occlusions and 39 with pulmonary embolism) were referred from the anticoagulant clinic to the thrombosis clinic for further evaluation and management. During the same time span another 168 consecutive pre-existing follow-up patients attended the same clinic for a total of 362 patients (**Table 1**). To validate the frequency of BD and its symptoms, we used a disease control group and a healthy control group as comparators. The disease control group derived from 127 consecutive patients attending a general haematology clinic and the normal control derived from 115 amongst friends and relatives of patients attending clinics and hospital personnel who self declared to be healthy. The survey was carried out according to the principles of the Declaration of Helsinki and verbal consent to participate was obtained by all participants^8^; the survey did not qualify as research because the questionnaire did not capture any personal identifiers hence no Ethics approval was required.^9^

**Table 1.** Demographics, signs, and symptoms of survey participants.

	**H-CTR**	**D-CTR**	**VTE**	
**No**	83	89	97	
**M/F**	31/52	46/43	44/53	
**Age (years±SD)**	42±10	42±9	40±8	
**Age at diagnosis (years±SD)**		33±13	32±14	
				
**BD**	1 (1.2%)	2 (2.2%)	1 (1.03%)	
				
**Not BD**	82	87	96	
	no (%)	no (%)	no (%)	
				
**Mouth ulcers**	5 (6.6)	10 (11.2)	5 (9.1)	ns
**Genital lesions**	2 (2.6)	0	2 (2)	ns
**Skin lesions**	6 (8)	9 (10.3)	14 (14.5)	ns
**Eye**	6 (8)	3 (3.4)	5 (9.1)	ns
**Joint**	10 (13.3)	12 (14.2)	14 (14.5)	ns
**Central nervous system**	6 (8)	4 (4.4)	4 (−3) (4.1)	ns
**Gastrointestinal**	4 (3)	7 (8)	9 (9.3)	ns
**Cardiovascular**	0	2 (2.2)	2 (2)	ns
**Lung**	3 (4)	1 (1.1)	1 (1)	ns
**Ear**	7 (8.5)	6 (6.8)	11 (11.4)	ns
**Exhaustion**	4 (3)	9 (10.3)	15 (15.6)	0.06
**Psychosis**	1 (1.3)	0	4 (4.1)	ns
**Familial BD**	0	0	0	
				
**Total**	49 (59.7)	63 (72.4)	86 (89.5)	p<0.0001

H-CTR: healthy controls; D-CTR: disease controls; VTE: venous thromboembolism; DD: disease duration; BD: Behçet’s disease.

### Questionnaire

**Figure 1.**
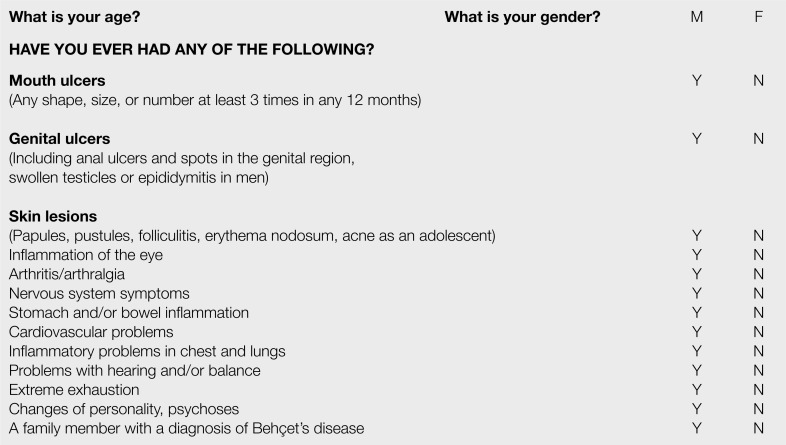
Clinical questionnaire on Behçet’s disease signs and symptoms.

The questionnaire shown in **Figure 1** was adapted from the International Criteria for Behçet’s Disease.^1^

### Inclusion and exclusion criteria

The inclusion criteria for the VTE group were: Caucasian patients between 18 and 45 years of age; documented VTE (deep or superficial veins) and or pulmonary embolism. The exclusion criteria were age range out-with 18 to 45 years, active or past history of solid cancer, acute and chronic renal failure, chronic hepatitis and/or liver cirrhosis, chronic infections, chronic inflammatory disorders (rheumatoid arthritis, psoriatic arthritis, ankylosing spondylitis, vasculitis, sarcoidosis, antiphospholipid syndrome, interstitial lung disease), insulin dependent and non-insulin dependent diabetes, porphyria. The same exclusion criteria were applied to the participants in the other two groups but with the addition of thrombosis.

### Statistics

Given a 7.5% prevalence of BD patients detected in a thrombosis clinic in Turkey (10) we argued that a sample size of 107 participants in each group had a 95% power to detect a significant difference with a 5% margin of error. Descriptive data are presented as percentages and mean ± standard deviations; frequencies between groups were compared by chi-square analysis.

## RESULTS

After application of the exclusion and inclusion criteria consecutive participants were invited to compile the questionnaire: 97 of 120 VTE (80%) patients agreed to do so as well as 89 of the 127 disease controls (70%) and 83 of the 115 healthy controls (72%). The study group suffered the following vascular occlusions: deep vein thrombosis in lower limbs n=72 (2 of which recurrent, 4 associated with pulmonary embolism, 1 with mesenteric vein occlusion, 1 with portal vein thrombosis); deep vein thrombosis in upper limbs n=2; pulmonary embolism n=16; isolated portal vein thrombosis n=2; cerebral sinus thrombosis n=5. The disease control group comprised patients with the following conditions: autoimmune haemolytic anaemia n=5, chronic immune thrombocytopenia n=14, chronic lymphocytic leukaemia stage A0 n=3, essential thrombocythemia n=5, primary eosinophilic syndrome (in remission) n=1, genetic haemochromatosis n=12, Hodgkin’s disease (all in remission) n=8, iron deficiency anaemia n=9, chronic immune thrombocytopenia n=16, monoclonal gammopathy n=4, follicular lymphoma n=6 (3 with indolent disease not requiring treatment, 3 in long standing remission), polycythaemia rubra vera n= 4. The average age, gender ratio and disease duration of the groups are shown in **Table 1**.

**Table 2.** Breakdown of signs and symptoms of survey participants.

	**H-CTR**	**D-CTR**	**VTE**
**Not BD**	82	87	96
	no (%)	no (%)	no (%)
			
**Mouth ulcers**	5 (6.6)	10 (11.2)	5 (9.1)
**Genital lesions**	2 (2.6)	0	2 (2)
Papules			
			
**Skin lesions**	6 (8)	9 (10.3)	14 (14.5)
Recurrent macules	3	5	4
Erythema nodosum	0	0	1
Psoriasis	1	1	2
Papular lesions	1	3	2
Fungus	0	0	2
Bullae	0	0	1
Eczema	0	0	1
Contact dermatitis	0	0	1
			
**Eye**	6 (8)	3 (3.4)	5 (9.1)
Dry eyes	2	1	1
Conjunctivitis	2	1	2
Blepharitis	1	1	0
Pterygium	0	0	1
Scleritis	0	0	1
Myopia	1	0	0
			
**Joint**			
Arthralgia	10 (13.3)	12 (14.2)	14 (14.5)
			
**CNS**	6 (8)	4 (4.4)	4 (4.1)
Chronic Headache	4	2	2
Epilepsy	0	1	1
Neuralgia	1	1	0
Depression	1	0	1
			
**GI**	4 (3)	7 (8)	9 (9.3)
Diarrhoea	1	1	2
Constipation	1	2	2
Haemorrhoids	1	1	1
Pancreatitis	0	1	0
Fatty liver	0	1	2
Cholecystitis	0	1	0
Cholelithiasis	0	0	1
Gastritis	1	0	1
			
**Cardiovascular**	0	2 (2.2)	2 (2)
Angina	0	0	1
Hypertension	0	1	1
NIDDM	0	1	0
			
**Lung**	3 (4)	1 (1.1)	1 (1)
Asthma	3	0	0
COPD	0	1	1
			
**Ear**	7 (8.5)	6 (6.8)	11 (11.4)
Tinnitus	2	0	1
Otitis media	0	1	2
Hearing loss	0	2	1
Vertigo	2	1	5
Glue ear	1	1	0
Meniere’s	0	1	2
Undefined	2	1	0

H-CTR: healthy controls; D-CTR: disease controls; VTE: venous thromboembolism; BD: Behçet’s disease; CNS: central nervous system; GI: gastrointestinal.

The criteria for BD were met by 1 participant in the VTE group, by 2 participants in the disease control group and by 1 participant in the healthy control group yielding a prevalence of 1.03%, 2.2% and 1.2% respectively (**Table 1**). The three patients in the diseased groups were referred to the rheumatology team who confirmed their diagnosis while the individual in the healthy group was not traceable given the anonymous nature of the questionnaire.

After excluding the patients diagnosed with BD from each group, participants from the VTE group reported more frequently exhaustion (15.6%), skin lesions and joint pains (14.5 %) ear problems (11%) and mouth ulcers (9%) (**Table 1**); although these frequencies were not different across the three groups, their sum significantly clustered in the VTE group as shown in **Table 1**. **Table 2** shows a detailed breakdown of signs of symptoms reported by all participants.

## DISCUSSION

By using a dedicated questionnaire, we found a 1.03% prevalence of BD in a series of consecutive British Caucasian patients with VTE; this is less than the 7.5% prevalence found in thrombotic patients attending a cardiovascular clinic from Turkey,^10^ where the prevalence of BD in the general population ranges between 8–42 per 100,000 inhabitants,^11^ much higher than the 0.27–1.47 prevalence for 100,000 inhabitants from UK, Germany and Sweden^3,12^; in this respect our 1.03% prevalence of BD is around 1000 fold that of the general UK population.^3,12^ It is very likely that the high prevalence of BD in Turkey plays a role in disease awareness not only amongst generalists but also amongst specialists dealing with thrombotic patients whereas, if it was not for our specific interest in the disease, we could have missed one BD patient every hundred patients with venous thrombosis. Indeed, the symptoms and signs of BD are often difficult to elicit from clinicians who have not had the chance to be exposed to such patients because of different expertise or training pathways: the detection of 2 attendees of the general haematology clinic fulfilling the criteria for BD favours this argument.

With regards to individual signs and symptoms not necessarily specific to BD in isolation, some, such as exhaustion, skin lesions and ear involvement, were more common in the VTE group: while fatigue is known to negatively predict physical and mental quality of life in patients with VTE,^13^ none of our patients had post-thrombotic syndrome with its skin signs^14^ and ear involvement was not present in the three patients with cerebral sinus thrombosis, making these associations purely casual.

Our survey has several weaknesses: 1) we did not have the chance to offer the questionnaire to a cohort of patients with thrombophlebitis that is another strong pointer for BD; 2) we could not offer a recall questionnaire to validate the responses as the questionnaire was answered in anonymity and no longer traceable to the person who completed it; 3) symptoms and signs were mostly self-reported, though it would not be different from how a clinician would carry out a clinical interview before examining a patient; 4) the average age of our participants was around 41±9 years whereas BD often develops between 20–40 years of age.^15^

In conclusion we found that BD may be under-diagnosed amongst attendees of a thrombosis clinic where instead it should be included in the differential diagnosis as the management of VTE in BD may deviate from the norm.^16^ Additionally, two BD diagnoses without VTE came from a general haematology clinic: perhaps posters in clinic rooms detailing the symptoms and signs of BD may raise awareness, hence diagnostic yields in unrelated specialties.
